# Cognitive Stimulation in Activities of Daily Living for individuals living with mild-to-moderate dementia (CS-ADL): a feasibility study

**DOI:** 10.12688/hrbopenres.14464.2

**Published:** 2026-10-15

**Authors:** Simone Ryan, Agnes Shiel, Orla Brady

**Affiliations:** 1 Discipline of Occupational Therapy, School of Health Sciences, University of Galway, Galway, County Galway, Ireland; 2 School of Allied Health, University of Limerick Faculty of Education and Health Sciences, Limerick, County Limerick, Ireland; 3 Occuaptional Therapy, Primary Care, Health Service Executive, HSE Dublin Mid Leinster, Trim, Co. Meath, Ireland

**Keywords:** Dementia; cognitive stimulation; occupational therapy; feasibility study; activities of daily living; ADL

## Abstract

**Background:**

Cognitive Stimulation in Activities of Daily Living (CS-ADL) is an occupational therapist-led group cognitive stimulation program for people living with mild-to-moderate dementia that aims to enhance functioning of activities of daily living (ADLs). This study investigated the feasibility of CS-ADL and a randomised controlled design comparing the effect of CS-ADL to treatment-as-usual (TAU) on ADL outcomes.

**Methods:**

This feasibility study used a non-randomiseddesign. Recruitment took place primarily within healthcare settings and supplemented via community outreach, media and voluntary organisations. Due to recruitment challenges, randomisation was not possible and between-group comparisons were not appropriate. ADL functioning, cognition, communication, quality of life (QOL) and neuropsychiatric symptoms (NPS) were assessed at baseline and follow-up. Data were analysed using descriptive statistics.

**Results:**

Sixteen participants were enrolled in the study, 12 of which completed follow-up assessments. Recruitment rates differed across intervention sites and affected the feasibility of study design. Thirteen participants were allocated to the intervention group (CS-ADL) and three were allocated to TAU. A reduction in caregiver distress from neuropsychiatric symptoms was observed in the intervention group at follow-up. No meaningful change at follow-up was identified in other secondary outcomes.

**Conclusions:**

This is the first study to evaluate the feasibility of a trial comparing CS-ADL to TAU. Several challenges were identified in relation to recruitment, study design and outcome measures that require modification to enhance feasibility and rigour of future trials. Preliminary results indicate CS-ADL may reduce caregiver distress related to neuropsychiatric symptoms. Further research is necessary to confirm these findings.

## Introduction

Dementia, now referred to as ‘major neurocognitive disorder’ ( American Psychiatric Association, 2013), is marked by a progressive decline in cognitive functioning that interferes with the performance of activities of daily living (ADLs), such as bathing, toileting, food preparation and home maintenance ( American Occupational Therapy Association, 2020). This functional decline increases dependency on care, as well as the risk of institutionalisation and mortality ( Mograbi et al., 2014). With the global cost of dementia in 2019 estimated at 1.3 trillion USD per annum ( World Health Organisation, 2017), and prevalence expected to undergo a threefold increase by 2050 ( Nichols et al., 2022), the development of effective interventions to address functional decline is essential.

Non-pharmacological interventions are increasingly utilised to address cognitive and behavioural outcomes of dementia ( Berg-Weger & Stewart, 2017). Cognitive stimulation is one such approach that aims to enhance global cognitive and social functioning by engaging participants in a range of stimulating and rewarding discussions and activities ( Clare & Woods, 2004). While various forms of cognitive stimulation exist ( Ryan & Brady, 2023), much of the research has focused on the manualised programme of Cognitive Stimulation Therapy (CST) ( Spector et al., 2003).

CST is a brief group intervention designed for individuals with mild-to-moderate dementia. CST consists of 14, 45-minute sessions delivered twice weekly over seven weeks, with many real-world applications incorporating the two 45-minutes sessions in one two-hour sessions, delivered once weekly. Intervention is based on eighteen fundamental principles which include mental stimulation, promoting new ideas, thoughts and associations, a focus on opinions rather than facts, maximising the potential of people with dementia, respect and person-centeredness ( Holden et al., 2020). CST has shown significant benefits for global cognitive functioning, quality of life, language and depressive symptoms ( Woods et al., 2023; Saragih et al., 2022). Several adapted formats have since been developed, including maintenance CST (MCST) which follows the initial programme ( Orrell et al., 2014), and individualised CST (iCST) delivered by caregivers ( Orrell et al., 2017).

While the primary outcome of cognitive stimulation is to enhance cognitive and social functioning, occupational therapists (OTs) frequently apply cognitive stimulation principles to promote health, wellbeing, and participation in everyday life. Moreover, CST is delivered by OTs as a means through which to remediate underlying impaired cognitive functions that impede occupational participation and ADL performance, otherwise termed a ‘bottom-up’ approach to intervention ( American Occupational Therapy Association, 2020). Their expertise in activity analysis and adaptation positions them uniquely to support individuals living with dementia to manage functional limitations (Association of Occupational Therapists Ireland, 2022). A recent systematic review and meta-analysis ( Woods et al., 2023) identified moderate- to high-quality evidence for cognitive stimulation in improving both ADL and IADL outcomes in mild-to-moderate dementia, suggesting that cognitive gains may transfer to functional improvements.

However, Woods et al.’s (2023) review included all formats of cognitive stimulation such as reality orientation, individualised interventions, and multicomponent interventions.

A recent umbrella review that focused exclusively on the effect of CST contrasted with Woods et al.’s (2023) review, reporting inconsistent findings across the literature regarding the effect of CST on ADL outcomes ( Cao et al., 2023). While CST has demonstrated effectiveness for a range of important cognitive and social outcomes, this uncertainty raises questions about the extent to which CST supports sustained occupational performance, and therefore its alignment with occupational therapy priorities. As engagement in occupation is a central goal of occupational therapy intervention in dementia care, intervention should demonstrate clear functional benefits to ensure optimal use of time and resources ( Walder et al., 2021).

In response to these findings, a scoping review was conducted to chart alternative formats of cognitive stimulation interventions that target functional outcomes for those living with dementia ( Ryan & Brady, 2023; *N* = 36). This review aimed to identify aspects of cognitive stimulation interventions that may support fucntional outcomes, complementing the aforementioned updated review of Woods et al. (2023). Findings of this review suggest that interventions incorporating the training and stimulation of ADLs may benefit functional outcomes. For instance, a pilot randomised controlled trial (RCT; *N* = 57) reported significant improvements in ADL performance following an occupational therapy program based on the training of ADLs through cognitive stimulation ( Palomares et al., 2021). These findings suggest task- or occupation-focused cognitive stimulation interventions may be effective in targeting functional outcomes and are more closely aligned with occupational therapy practice.

Building on this evidence, CS-ADL (Cognitive Stimulation in Activities of Daily Living) was developed. CS-ADL is as an occupational therapy group cognitive stimulation intervention for individuals living with mild-to-moderate dementia. CS-ADL aims to enhance ADL performance, alongside social and cognitive functioning through the stimulation and practice of everyday activities. The CS-ADL programme was developed scientifically based on the currently available literature and refined after a sample format was tested in an Irish healthcare setting, and evaluated qualitatively ( Ryan, Chockalingam, & Brady 2024a). CS-ADL reflects previous similar cognitive stimulation interventions that have demonstrated benefits for functional outcomes ( Palomares et al., 2021; Luttenberger et al., 2012; Ryan & Brady, 2023) while maintaining a format comparable to CST, which is widely implemented within clinical practice ( Kelly et al., 2024). It primarily differs to CST with regards to its focus on ADL practice and ADL-themed cognitive stimulation activities, further discussed in the methods section.

A qualitative study (n = 6) of a pilot of CS-ADL demonstrated its acceptability among people living with dementia and their caregivers, with perceived benefits for memory, mood, communication, and social connectedness ( Ryan, Chockalingam & Brady, 2024a). However, limited data were gathered regarding the effect of CS-ADL on ADL outcomes, necessitating further research. Randomised controlled trials (RCTs) are widely regarded as the gold standard for evaluating intervention effectiveness. However, RCTs are often resource- and time-consuming in nature. Therefore, a feasibility study is a necessary preliminary step to assess the practicality and acceptability of both the CS-ADL intervention and the proposed study design prior to large-scale evaluation. In addition, this study aimed to explore post-intervention changes in ADL functioning, cognition, communication, neuropsychiatric symptoms (NPS) and quality of life (QOL).

## Methods

### Study design

A non-randomised, feasibility design was conducted. The study ran from November 2023 to June 2024. This study design was chosen as no prior studies had evaluated the current CS-ADL intervention with regards to optimal content and delivery; it was anticipated that further intervention refinements would be required prior to future pilot trials and larger-scale evaluation ( Skivington et al., 2021).

While planned, randomisation and between-group comparisons were not feasible due to slow and differing rates of recruitment across study sites, which will be discussed further in the results and discussion sections. Participants were instead non-randomly allocated to either intervention (CS-ADL) or control conditions (TAU). It was not possible to blind participants to treatment allocation due to the nature of intervention. Blinding of outcome assessors was not feasible for this study due to resource and practical constraints.

### Participant sample

Participants were eligible for inclusion if they had a formal diagnosis of major neurocognitive disorder (dementia) ( American Psychiatric Association, 2013) and mild-to-moderate cognitive impairment classified by the Montreal Cognitive Assessment (MoCA; ≥ 10) ( Nasreddine et al., 2005). Participants were required to have some ability to communicate and understand communication, determined by a score of 0 or 1 on questions 12 and 13 of the Clifton Assessment Procedures for the Elderly-Behaviour Rating Scale ( Pattie & Gilleard, 1975). Exclusion criteria included the presence of pre-morbid intellectual disability, or significant visual/hearing impairment that would interfere with participation in a group intervention. Individuals with significant physical illness/disability that would interfere with group participation were excluded i.e. medical instability, terminal illnesses, severe cardiac or respiratory conditions, or conditions that severely limit mobility. Individuals with uncontrolled disruptive behaviours that would interfere with group participation, such as aggression, were also excluded. This criterion was assessed informally by gatekeepers using clinical reasoning. Participants taking dementia medication continued to do so during the study. As several outcomes were proxy-rated, caregivers were required to be available during data collection procedures.

Hypothesis testing was not an objective of this feasibility study; therefore, formal power calculations are not appropriate. However, a target sample size of 34 participants was chosen in line with previous cognitive stimulation intervention pilot research ( Dolan et al., 2022) and a review of sample sizes for UK pilot and feasibility studies ( Totton et al., 2023). This sample size was pragmatic and based on anticipated intervention group sizes within intended randomisation.

### Recruitment and consenting procedures

Community-dwelling individuals with dementia were initially recruited from primary care, memory technology resource rooms (MTRR), and psychiatry of later life services across four counties in Ireland. Where challenges with recruitment arose, additional ethical approval was obtained to supplement recruitment through the Alzheimer Society of Ireland (ASI), outreach events, print and radio advertisements.

The consent process for this study aligned with Irish capacity legislation (Assisted Decision-Making (Capacity) Act, 2015), the Health Service Executive (HSE) National Consent Policy (2022) and the Data Protection Act 2018 Health Research Regulations (Government of Ireland, 2018). Written informed consent was sought from the individual with dementia to participate in the study with the automatic presumption of capacity. While it is possible that individuals contacted regarding the study had challenges in their ability to understand, retain, use or weight, and communication information during the decision-making process, it was not within the scope nor ethical remit of this study to request a functional capacity assessment related to the individual’s decision to participate in the research. If an individual refused consent to participate, no further actions were taken to uphold and respect their dignity and autonomy.

Nevertheless, all actionable steps were taken to support the individual’s decision-making process. Gatekeepers familiar with the prospective participants provided study information verbally, and enquired whether participants wished to contact the researcher themselves, or whether they would prefer for the researcher to contact them directly. For the latter, verbal consent was obtained for participant contact details to be provided to the researcher for the sole purpose of providing further study information. Study information was initially provided verbally over phone call, and participant information leaflets were thus provided to support the decision-making process, with the individual’s caregiver informed of the research where possible. All participant-facing documentation followed dementia-friendly document guidelines. Continued assent was not formally assessed; however, the researcher informally assessed verbal and non-verbal participant cues throughout the study and ensured to continually inform participants and caregivers that they could withdraw from the study at any stage prior to data anonymisation.

### Outcomes and data collection procedures

Primary outcomes of this study related to various feasibility indicators (recruitment, retention, adherence, intervention integrity, adverse events). Acceptability of CS-ADL has been evaluated previously ( Ryan, Chockalingam & Brady, 2024a). The total number of people screened and recruited to the study were recorded, alongside the total number of dropouts and their reasons. The number of intervention sessions each participant attends, as well as the number of adverse events that occurred, were recorded. An intervention manual was developed by the research team to support standardised delivery across sites; any deviations from the intervention manual were recorded to capture intervention integrity and were reflected on after each intervention session.

Secondary outcomes of this study related to intervention outcomes, including ADL functioning, cognition, QOL, communication, neuropsychiatric symptoms, and occupational performance within a group setting. While this study did not intend to produce a definitive evaluation of the effect of CS-ADL on these outcomes, it is important to assess intervention outcomes in feasibility studies to evaluate chosen measurement tools and the potential for the intervention to affect likely outcomes in future trials. Secondary outcomes were measured in-person at baseline and within one month of study completion. Data were collected at recruitment sites by the principal investigator.

ADL functioning was measured using the caregiver-rated Alzheimer’s Disease Cooperative Study-Activities of Daily Living (ADCS-ADL) scale ( Galasko et al., 1997). The ADCS-ADL is a 23-item proxy-rated questionnaire evaluating six basic and 17 instrumental ADL outcomes. Total score ranges from 0–78, with higher scores indicating greater independence and lesser deficit. The ADCS-ADL was chosen as it is disease-specific and comprehensively addresses all relevant ADL outcomes for people living with dementia. It has also demonstrated high internal consistency (Cronbach’s α = 0.91) and high sensitivity to functional changes in mild-to-moderate dementia.

Cognition was measured using the Repeatable Battery for Assessment of Neuropsychological Status (RBANS) ( Randolph et al., 1998), assessing immediate memory, visuospatial/constructional ability, language, attention and delayed memory. The RBANS is a neuropsychological battery consisting of 12 subtests producing individual scaled scores for each subtest, and a total scaled score. Higher scores indicate lesser deficits. Alternate forms of the RBANS were used pre- and post-test to minimise practice effects. The RBANS has good reliability and adequate validity indicators, with high sensitivity (90%) and specificity (90%) in dementia. As blinding was not possible for this outcome domain, standardised manual instructions were followed to minimise bias.

QOL was measured using the Quality of Life-Alzheimer’s Disease (QOL-AD) ( Logsdon et al., 1999). The QOL-AD is a thirteen-item scale rated by both participants and caregivers. Each item is rated on a four-point Likert scale. Total score of self-report and proxy-ratings are combined, with the total score ranging from 26–104. Higher scores indicate greater quality of life. The QOL-AD has demonstrated good content and construct validity, inter-rater reliability (Cohen’s ĸ > 0.70) and test-retest reliability, with excellent internal consistency (Cronbach’s α = 0.82). It correlates well with the Dementia Quality of Life scale (0.69) and with the EuroQol-5D scale (0.54), indicating good criterion concurrent validity. The QOL-AD is recommended to measure QOL in psychosocial intervention research in dementia care ( Moniz-Cook et al., 2008).

Communication was measured using the caregiver-rated Holden Communication Scale (HCS). The HCS is a 12-item proxy-rated scale. There are five response options per individual item, ranging from zero to four. Total score ranges from 0–48, with higher scores indicating greater deficit. The HCS is commonly used to evaluate communication outcomes of cognitive stimulation interventions ( Woods et al., 2023) and has demonstrated good psychometric properties for people with dementia, with high internal consistency (Cronbach’s α = 0.94) and test-retest reliability (r = 0.71).

Severity of NPS and associated caregiver distress were measured using the caregiver-rated Neuropsychiatric Inventory-Questionnaire (NPI-Q). The NPI-Q is a 12-item proxy-rated questionnaire, with each item rated ‘yes’ or ‘no’ to the presence of NPS. If the NPS is present, severity of NPS is rated on a 3-point scale and associated caregiver distress is rated on a five-point scale. Total severity score ranges from zero to 36, and total caregiver distress score ranges from zero to 60. Higher scores indicate both greater severity and distress. The NPI-Q has demonstrated adequate test-retest reliability, convergent validity and internal consistency, and is suitable for measuring mood and depressive symptoms in dementia patients.

Occupational performance within a group setting was measured within the treatment group using the Occupational Therapy Task Observation Scale (OTTOS) ( Margolis et al., 1996). This is a 15-item observational scale, with 10 items evaluating task functions and five items evaluating general behaviour. Each item is rated on a 10-item Likert scale, with higher score indicating lesser deficit. The OTTOS has demonstrated High interrater reliability for task behaviour scores (r = 0.89), general behaviour scores (r = 0.76) and total scores (r = 0.92). The OTTOS was used as a supplementary measure to aid interpretation of the overall effect of CS-ADL on everyday functioning.

### Treatment conditions

The intervention arm received CS-ADL, an occupational therapist-led, group cognitive stimulation program that has been described elsewhere ( Ryan, Chockalingam & Brady, 2024a). An overview of the weekly session plans is provided in Table 1. In brief, CS-ADL is a multi-component group programme, with each session consisting of cognitive stimulation, physical activity and ADL practice. Sessions involve stimulation of a range of ADLs including dressing, grooming, meal preparation, laundry and leisure. CS-ADL sessions lasted approximately two hours and were delivered once weekly for seven weeks at two centres. The principal investigator delivered the intervention independently at one site due to practical constaints, with assistance from occupational therapy and nursing doctoral students at the second site. Intervention was delivered in the occupational therapy kitchen and ADL room at each site, with an OT kitchen, dining, bed and bathroom area in situ at each centre. A separate room was made available at the second site for caregivers of CS-ADL participants; this was not provided at the first site due to space constraints.

**Table 1.** CS-ADL session overview.

Session	Theme	Warm-up activities	Cognitive stimulation activities	ADL practice
1	Morning routine	Greetings, group song, RO. Physical game e.g. balloon tennis.	Identification and categorisation of morning routine-related items. Discussion and demonstration of assistive devices. Discussion and reminiscence of morning routine and breakfast habits.	Preparation of a hot breakfast.
2	Afternoon routine	Greetings, group song, RO. Physical game e.g., skittles.	Discussion and demonstration of getting out of bed, getting up from a fall and relevant assistive devices. Discussion and reminiscence of afternoon routine. Discussion and demonstration of to-do lists and reminders.	Mannequin dressing activity. Simple gardening activity.
3	Domestic activities 1	Greetings, group song, RO. Physical game e.g., cue card game.	Discussion, reminiscence and sequencing of household chores. Identification and categorisation of chore-related items. Sensory-based activity: household scents.	Clothesline activity*. Preparation of a sandwich.
4	Evening routine	Greetings, group song, RO. Physical game e.g., target game.	Discussion and reminiscence of evening and dinner routine. Identification and categorisation of dinner-related items. ADL charades game. Discussion and demonstration of evening routine-related assistive devices.	Preparation of a dinner.
5	Domestic activities 2	Greetings, group song, RO. Physical game e.g., balloon tennis.	Discussion and reminiscence of grocery shopping. Numbers-based grocery activity. Discussion and demonstration of diaries.	Preparation of lemonade.
6	Baking	Greetings, group song, RO. Physical game e.g., seated hockey game.	Discussion, reminiscence and sequencing of baking. Identification and categorisation of baking-related items.	Preparation of scones. Cleaning of baking materials.
7	Entertainment and leisure	Greetings, group song, RO. Physical game e.g., skittles.	Discussion and reminiscence of leisure, music, sport using a presentation Presentation/quiz of famous musicians, actors and athletes.	Meaningful leisure activity e.g. card games, painting, music.

The control arm of the study received TAU. Participants received TAU at their respective clinical setting where recruitment took place and consisted of the routine care that participants received from the health services. TAU may have differed between participants and was determined by healthcare providers who are external to the research. It may have included medication, occupational therapy, physical therapy, nursing or attendance at a day care centre. It may also have consisted of no active treatment or service. It was not possible for participants in the control to receive CS-ADL as part of their usual care as it was not implemented in the services where participants were recruited from. At follow-up, control group participants were offered a home programme with cognitive stimulation resources, designed to be completed jointly by the person with dementia and their caregiver in the home environment, with training provided by the researcher at follow-up. This home programme consisted of similar content to CS-ADL intervention sessions, with recommendations and resources on various compensatory strategies, environmental modifications to enhance participation and cognitive stimulation in everyday activities.

### Analysis

Participant characteristics were analysed using descriptive statistics via Statistical Package for Social Sciences (SPSS) version 28.00. A CONSORT flow diagram was used to present information relating to the feasibility indicators of recruitment and retention. Feasibility outcomes were summarised using descriptive statistics. Continuous data were assessed for normality using the Shapiro-Wilks test, with means and standard deviations (SD) reported where data were normally distributed. 

### Ethical approval and informed consent statements

Ethical approval was obtained from the Health Service Executive North East Research Ethics Committee (REC/23/042), the Reference Research Ethics Committee Midlands Area and Corporate (RRECB1223SR), and the Saolta Clinical Research Ethics Committee (C.A. 3138). The consent process for this study adhered to the Irish Assisted Decision-Making (Capacity) Act (2015). Written informed consent was obtained from each participant.

## Results

### Recruitment. randomisation and retention

Thirty individuals were screened, with 16 participants enrolled in the study during the study period (November 2023–June 2024). Recruitment ceased due to time constraints. Reasons for non-enrolment included no formal diagnosis of dementia (n = 5; 16.67%), presence of severe cognitive impairment (n = 1; 3.33%), and self-withdrawal due to medical instability, onset of uncontrolled neuropsychiatric symptoms, change of interest, or participant self-reported feelings of unsuitability for intervention (n = 8; 26.67%).

Recruitment windows differed across sites due to delays in ethical approval. Figure 1 provides a breakdown of recruitment windows at each site, with further detail of the source of participant referral to the study.

**Figure 1.**
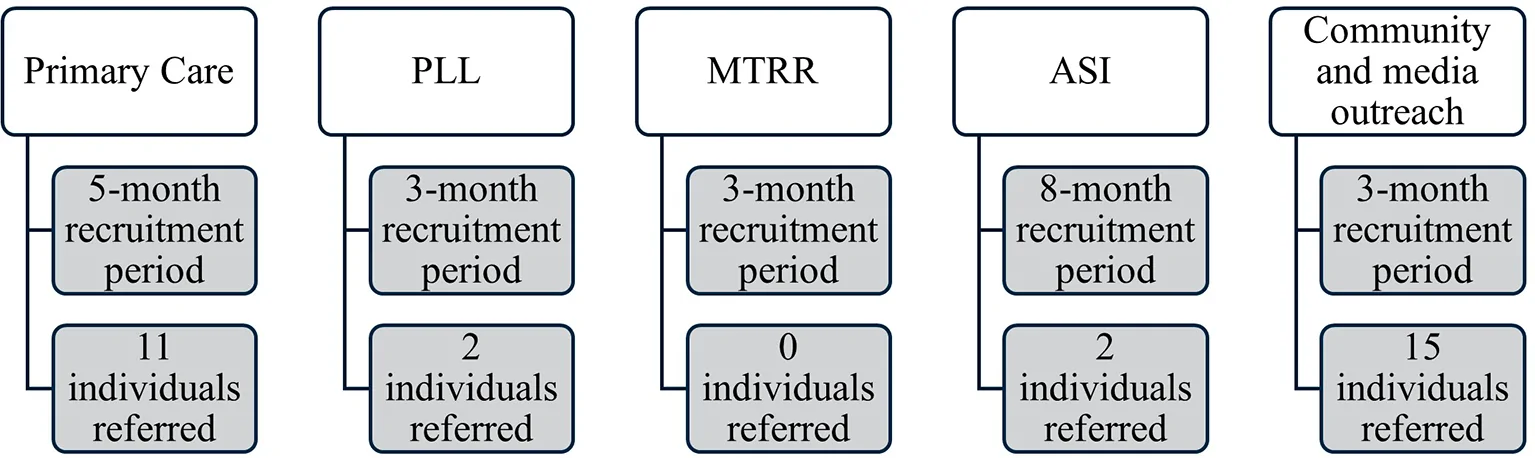
Results of recruitment at each site.

It was initially planned to randomise participants into intervention and control conditions at each site; however, randomisation was not feasible due to the slow and differing rates of recruitment across study sites. Ultimately one site was non-randomly allocated as the control condition as there were insufficient numbers to randomise within each recruitment site, nor to complete cluster randomisation. Recruitment was ultimately ceased due to time constraints.

Thirteen participants were thus allocated to the treatment group and three to the control group. One participant allocated to the treatment condition withdrew following baseline assessment, and another was lost to follow-up. In the control condition, it was not possible to complete follow-up with one participant due to logistical challenges, and another was lost to follow-up due to hospital admission, resulting in a total dropout rate across both study arms of 6.25%.

A CONSORT flow diagram ( Eldridge et al., 2016) depicts the flow of participants through the study ( Figure 2).

**Figure 2.**
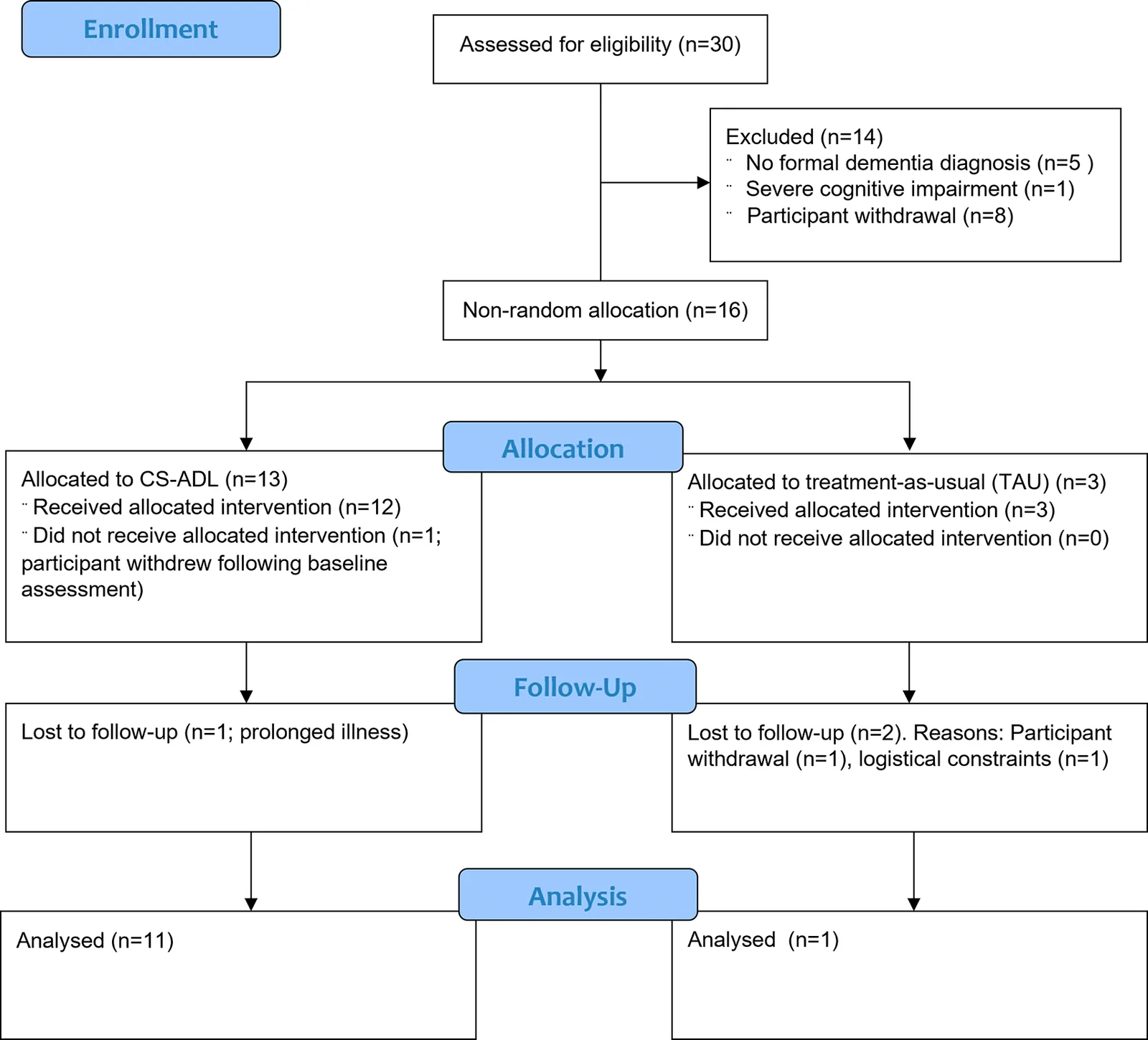
CONSORT flow diagram.

### Participant characteristics

The mean age of the sample was 77.4 years, with eleven women and five men enrolled in the study. Most study participants reported a diagnosis of Alzheimer’s disease (n = 6; 37.5%). Remaining participants reported a diagnosis of vascular dementia (n = 2; 12.5%), mixed dementia (n = 1; 6.25%), frontotemporal dementia (n = 1; 6.25%), primary progressive aphasia (n = 1; 6.25%) and an unspecified aetiology (n = 5; 31.25%). The mean MoCA score was 20.27 (SD 4.5). All participants were of Irish ethnicity, with only two participants living independently. Comparative analyses were not performed due to sample size disparity. Further demographic details of study participants are provided in Table 2.

**Table 2.** Participant characteristics.

Characteristics	Treatment group ( *n* = 13)	Control group ( *n* = 3)
Mean age in years (SD)	77.69 (7.9)	76.3 (3.2)
MoCA mean score (SD)	18.69 (4.6)	22 (3.6)
Gender, n (%) Male Female	5 (38.5) 8 (61.5)	0 (0) 3 (100)
Dementia type, n (%) Alzheimer’s disease Vascular dementia Mixed dementia FTD PPA Unspecified	5 (38.5) 1 (7.7) 1 (7.7) 1 (7.7) 1 (7.7) 4 (30.8)	1 (33.3) 1 (33.3) 0 (0) 0 (0) 0 (0) 1 (33.3)
Medication type, n (%) Rivastigmine Memantine Donepezil Unspecified Multiple reported None	1 (7.7) 3 (23.1) 2 (15.4) 3 (23.1) 1 (7.7) 3 (23.1)	0 (0) 2 (66.7) 0 (0) 1 (33.3) 0 (0) 0 (0)
Living arrangements, n (%) Lives with spouse or relative Lives alone	14 (87.5) 2 (12.5)	3 (100) 0 (0)
Ethnicity, n (%) White Irish	13 (100)	3 (100)

**MoCA**: Montreal Cognitive Assessment; **FTD:** Frontotemporal dementia; **PPA:** primary progressive aphasia.

### Outcomes

Due to the difference in sample size across study arms, differences between groups at follow-up were not analysed. Table 3 details the treatment group’s baseline and follow-up outcomes. Table 4 outlines the sole control group participants results.

**Table 3.** Treatment group results.

Outcome tool	Treatment group ( *n* = 11)		
	Baseline mean (SD)	Follow-up mean (SD)	Mean difference (95% CI)
ADCS-ADL	54.18 (7.2)	54.36 (9.2)	0.18 (−3.21, 3.58)
QOL-AD	66.0 (13.1)	64.2 (14.0)	0.18 (−3.21, 3.58)
HCS	12.64 (5.0)	12.64 (5.6)	0.00 (−1.78, 1.78)
NPI-Q severity	8.10 (6.1)	7.64 (4.0)	−0.45 (−3.06, 2.15)
NPI-Q caregiver distress	8.69 (9.1)	6.73 (6.8)	−2.82 (−5.52, −0.12)
RBANS (total) Immediate memory Attention Language Visuospatial/constructional Delayed memory	56.3 (10.65) 55.70 (13.3) 70 (15.6) 71.10 (20.0) 63.92 (11.2) 52.40 (13.8)	59.1 (10.8) 60.70 (12.1) 75.2 (13.8) 75.3 (18.1) 64.5 (14.6) 54.60 (13.9)	2.80 (−3.55, 9.15) 5.00 (−3.76, 13.76) 4.70 (−6.22, 15.62) 4.20 (−1.85, 10.25) −0.10 (−8.55, 8.35) 2.20 (−5.61, 10.01)
OTTOS	150 (20.91)	152.91 (23.35)	2.22 (−11.16, 15.61)

**SD** **:** Standard deviation; **CI:** Confidence interval; **ADCS-ADL:** Alzheimer’s Disease Cooperative Study-Activities of Daily Living; **QOL-AD:** Quality of Life-Alzheimer’s Disease; **HCS:** Holden Communication Scale; **NPI-Q:** Neuropsychiatric Inventory-Questionnaire; **RBANS:** Repeatable Battery for Assessment of Neuropsychological Status **; OTTOS:** Occupational Therapy Task Observation Scale.

**Table 4.** Control participant results.

Outcome tool	Baseline score	Follow-up score	Change in score
ADCS-ADL	57	63	+6
QOL-AD	84	81	−4
HCS	6	10	−4
NPI-Q severity	0	0	0
NPI-Q caregiver distress	0	0	0
RBANS Immediate memory Attention Language Visuospatial/constructional Delayed memory	81 61 115 85 121 48	75 65 112 82 100 40	−6 +4 −3 −3 −21 −8

**ADCS-ADL:** Alzheimer’s Disease Cooperative Study-Activities of Daily Living; **QOL-AD:** Quality of Life-Alzheimer’s Disease; **HCS:** Holden Communication Scale; **NPI-Q:** Neuropsychiatric Inventory-Questionnaire; **RBANS:** Repeatable Battery for Assessment of Neuropsychological Status.

For cognitive domains, while confidence intervals were wide and crossed the null suggesting imprecision, magnitude of the mean differences may indicate a potential beneficial effect of intervention. For instance, improvements were observed in RBANS total score (mean difference = 2.80, 95% CI −3.55 to 9.15), immediate memory (mean difference = 5.00, 95% CI −3.76 to 13.76), attention (mean difference = 4.70, 95% CI −6.22, 15.62), language (mean difference = 4.20, 95% CI −1.85, 10.25), delayed memory (mean difference = 2.20, 95% CI −5.61, 10.01).

A reduction in caregiver distress as measured by NPI-Q was observed (mean difference = −2.82, 95% CI −5.52 to −0.12).

For several outcomes, no meaningful change from baseline to follow-up was identified within the treatment group: ADCS-ADL (mean difference = 0.18, 95% CI −3.21 to 3.58); HCS (mean difference = 0.00, 95% CI −1.78 to 1.78); QOL-AD (mean difference = −1.80, 95% CI −6.31 to 2.71); NPI-Q symptom severity (mean difference = −0.45, 95% CI −3.06 to 2.15); OTTOS (mean difference = 2.22, 95% CI -11.16, 15.61).

### Intervention adherence

The mean attendance for CS-ADL sessions was 5.75 out of 7. Reasons for non-attendance included illness, mood instability, forgetting, or conflict with medical appointments. No dropouts occurred after intervention commencement.

### Intervention integrity

Intervention integrity was maintained across study sites concerning the implementation of essential content of CS-ADL. Some activities were adapted to ensure appropriate engagement of all participants; this is inherent to the principles of cognitive stimulation and CST ( Spector et al., 2020). For example, modification was made to the ADL practice component of session six (theme: ‘Baking) of one CS-ADL group as there was an unequal balance of men and women in this group. It was decided to allow participants to choose between baking and a DIY activity to accommodate for meaningful engagement of each gender. Despite this modification, the core function and purpose of CS-ADL remained intact and is within the parameters of intervention fidelity.

Adherence to the intervention protocol was impacted by the availability of equipment and resources at each site. Furthermore, timing of sessions impacted adherence to the intervention protocol; it was ensured that sessions did not exceed the two-hour timeframe, meaning that some activities had to be excluded. Any excluded activities were later reintroduced in following sessions when time allowed. Reality orientation activities were also excluded from one CS-ADL group following participant requests to discontinue these activities as the purpose was unclear, and it was not deemed relevant or meaningful to them.

### Adverse events

One adverse event was reported in the intervention group; one participant choked on food prepared in an intervention session. The Heimlich manoeuvre was performed and was successful. An incident report form was filed, and a speech and language therapy referral for review of the participant’s swallow was completed. The participant’s caregiver was informed.

## Discussion and implications

This study aimed to evaluate the feasibility of CS-ADL and the study design of an RCT comparing CS-ADL to TAU for people living with mild-to-moderate dementia and to collect preliminary evidence regarding the effect of CS-ADL on participants’ ADL functioning, cognition, communication, QOL, NPS and occupational performance.

### Discussion of trial feasibility

Recruitment to this study proved challenging, failing to meet the proposed sample size within the study timeframe. Recruitment difficulties in non-pharmacological dementia trials are well-documented ( Field et al., 2019). Strict eligibility criteria and the high prevalence of underdiagnosis in community services compounded these challenges ( Beattie et al., 2018; Begley et al., 2023). However, restrictive inclusion criteria ensure scientific rigour.

Healthcare professionals’ competing priorities further hindered recruitment, a common issue in under-resourced health systems as observed in the Irish health service ( Fleming et al., 2022). Additionally, short, inconsistent recruitment windows across sites approval may have exacerbated recruitment challenges. This was due to delays in ethical approval; the present ethical review process in Ireland involves several dozen committee operating at a local or institutional level. Applications were made to three separate ethical committees for approval of this study, with inconsistencies noted across each submission and review process, subsequently impacting the timeline of the study. However, supplementary recruitment strategies used in this study, such as community outreach, patient registries, and media engagement yielded successful results.

Caregiver involvement in data collection may have impeded recruitment, as caregivers may perceive research participation as an additional burden ( Whitebird et al., 2011). Provision of incentives can help minimise this burden. For instance, room space and refreshments were provided to caregivers of one intervention group, potentially alleviating burden in relation to travel and offering peer support opportunities.

The study’s non-randomised design was a deviation from initial study protocol due to recruitment challenges. It was initially intended to randomise participants to treatment and control conditions within each distinct site. However, insufficient participants were recruited at each site for randomisation to be undertaken, as CS-ADL is a group intervention and required a minimum number of participants for the intervention to be delivered. Cluster randomisation was considered as an alternative randomisation procedure, where study sites would be randomised to treatment conditions, as opposed to individual participants. However, this was ultimately not feasible in the current study, due to differing rates of recruitment at each planned cluster site and time constraints. Instead, participants were non-randomly allocated depending on their intervention site. Due to differing rates of recruitment, this resulted in low numbers within the control group.

The practical constraints of randomisation in clinical research have been noted previously in the literature ( Colliver & McGaghie et al., 2008). While non-randomised designs, as ultimately used in the current study, are pragmatic, they introduce biases and confounds that threaten study validity ( Colliver & McGaghie et al., 2008). Cluster randomisation is commonly used to evaluate complex health interventions, enhancing the practicality of large-scale research. Previous non-pharmacological dementia intervention trials, including cognitive stimulation, have demonstrated the feasibility of this approach ( Jøranson et al., 2015; Mraz, Tong & Liu, 2023); however, cluster randomisation was not successful in the current study. Use of a stepped-wedge cluster randomised design may be of greater applicability for this research, whereby cluster groups cross over from the control to the intervention conditions. While there are practical and statistical complexities to this approach, it has been tested in previous cognitive stimulation research and found to be both feasible and acceptable (Paddick et al. 2017). Future trials of CS-ADL may consider use of this design to enhance feasibility and maximise recruitment and retention of control participants, particularly in regions where participants have limited access to dementia services.

Intervention feasibility varied by site. Concerns regarding the safety and quality of facilitation arose when only one facilitator was present. Some participants needed extra encouragement or activity adaptation, which was challenging for a sole facilitator to undertake successfully. These issues were mitigated with additional co-facilitators. A minimum of two facilitators is thus recommended for CS-ADL groups larger than three participants, aligning with guidance for the widely implemented CST. While CS-ADL must be led by an occupational therapist, co-facilitators can be from other professions like nursing or psychology, as intervention may also align with the service goals of these professions.

Intervention fidelity concerns emerged when session components were omitted due to time or resource constraints. Participant feedback at one site also led to the exclusion of reality orientation activities, as they felt activities were irrelevant to their daily lives. Reality orientation has previously been suggested to cause frustration in dementia ( Savorani et al., 2004), with CST guiding principles advising to use orientation sensitively and implicitly. Reconfiguration of the intervention content regarding orientation, as well as intervention structure and timings is recommended to maintain fidelity in future trials. However, direct observation of fidelity would have strengthened fidelity assessments in this study and should be considered in future trials. 

### Discussion of study outcomes

Comparing outcomes between intervention and control groups was not possible. Within the intervention group, CS-ADL did not demonstrate a meaningful improvement in ADL functioning, a contrast from similar multi-faceted cognitive stimulation interventions ( Palomares et al., 2021). This may be attributed to the small sample size. Furthermore, reliance on within-group analyses limits the strength of conclusions; cognitive stimulation has been shown to contribute to a delay in deterioration when compared to a no treatment control ( Chapman et al., 2004).

In addition, the ADCS-ADL may not align with the aim of CS-ADL; it is possible that CS-ADL does not contribute to direct improvements in ADL performance, but rather to an enhancement of occupational engagement and participation, as previous qualitative research suggests ( Liu et al., 2020). Few measures of everyday occupational engagement and participation in dementia exist, thus qualitative evaluation may better capture the subjective dimensions of these constructs.

While improvements were observed in several cognitive domains within the treatment group, results are imprecise and uncertainty remains. These findings contrast with previous research that shows small, short-term cognitive benefits from cognitive stimulation ( Woods et al., 2023). The RBANS may be confounded by the tool’s dependence on language comprehension and expression, abilities often impaired in dementia. Furthermore, neuropsychological measures may not be the most appropriate for evaluating the effect of CS-ADL on cognitive abilities, as they are not reflective of everyday performance. CS-ADL targets cognitive functions such as initiation, organisation, and planning of everyday activities; processes that underpin the construct of ‘functional cognition’. Measures of functional cognition may thus be more appropriate in future research.

While no changes in NPS severity were found, a reduction in caregiver distress due to NPS was observed. This finding is supported by previous research where caregivers report lower burden following their care-recipient’s participation in cognitive stimulation ( Farina et al., 2007). Providing room space to caregivers might have alleviated distress through peer-support opportunities. As caregiver wellbeing impacts the quality of care provided to persons living with dementia, future implementation of CS-ADL should consider the provision of opportunities for caregiver peer-support, which may in turn enhance recruitment and retention.

No changes in QOL or communication were identified post-intervention. These findings contrast with literature ( Ryan, Chockalingam & Brady, 2024a; Woods et al., 2023; Chao et al., 2020). Outcome measures may have been insensitive to post-intervention changes. In addition, reduced quality of intervention facilitation at one site may have impacted participant engagement, thus affecting gains in communication. Ensuring an appropriate therapist-participant ratio is crucial for future research.

Lastly, no changes in occupational performance were identified. Several OTTOS items including ‘engagement’, ‘quality of work’ and ‘activity level’ often depended on participants interest and prior experience with intervention activities. Participants lacking prior experience or interest may thus have received lower OTTOS ratings, possibly distorting overall group mean. Additionally, factors like fluctuations in mood, poor sleep and physical health impacted performance. Future trials should consider alternative observational tools validated for dementia.

### Limitations

The study’s validity and generalisability are limited by several factors. Outcomes were assessed by the intervention facilitator, and thus not blinded to treatment allocation, risking detection bias. This was due to resource constraints common within feasibility studies, and future trials will ensure blinding of outcome assessors to enhance validity of findings. The sample size was not reached, limiting analyses and conclusions regarding CS-ADL’s effect on study outcomes are therefore limited. However, the primary aims of this study were to evaluate the feasibility of the methodology, therefore findings of this study are not anticipated to be confirmatory. In addition, the lack of Public-Patient-Involvement (PPI) is a recognised limitation of this study, as PPI involvement may have enhanced the relevance of the research question and accessibility of recruitment and intervention procedures. It is acknowledged that variation in co-facilitators available to support intervention at each site may have influenced overall feasibility and intervention outcomes, as discussed above. However, this was due to practical and resource constraints and provides valuable insights for feasibility of intervention delivery for future trials and implementation.

### Implications for future practice and research

This research furthers the evidence-base for CS-ADL and similar occupational therapy-led cognitive stimulation interventions. However, further development and evaluation of CS-ADL is required prior to a definitive trial, as several uncertainties remain in relation to outcome assessment, study design and intervention, therefore requiring further evaluation.

The challenges encountered in recruitment and retention highlight the need for careful consideration in the design of future trials evaluating CS-ADL in people living with mild-to-moderate dementia. Future research should ensure a comprehensive recruitment strategy, with consideration of alternative methods of randomisation to enhance feasibility, for example cluster randomisation.

Public-Patient-Involvement (PPI) should be integrated within the design and conduct of future CS-ADL research to aid development of recruitment and retention strategies, advise on study design, and oversee modification of intervention protocol to ensure meaning and relevance to the participant population.

Alternative methods of data collection are also recommended for future research, with consideration of measures of functional cognition and occupational engagement, alongside consideration of a mixed methods approach to evaluation to enhance understanding of the effects of intervention.

## Conclusions

Significant recruitment challenges indicate a need for study design modifications to enhance future trial feasibility. This study did not identify effects of CS-ADL on ADL functioning, communication, QOL, occupational performance, or NPS severity. However, it did identify a significant reduction in caregiver distress related to dementia neuropsychiatric symptoms and some potential benefits for cognition. However, these findings are not confirmatory due to the small sample size; instead, these findings provide preliminary evidence for CS-ADL and highlight implications for future evaluation and implementation of CS-ADL. These findings are of relevance to occupational therapists implementing cognitive stimulation in their therapeutic practice.

## Consent

Written informed consent was obtained by the participants.

## Data Availability

The underlying anonymised study data are available under the terms of the Creative Commons Attribution 4.0 International license (CC-BY 4.0). Data has been deposited in Figshare: Dataset CS-ADL pilot trial https://doi.org/10.6084/m9.figshare.26502529 ( Ryan, Shiel & Brady, 2024b).
