# Supplementary material for: Cognitive Stimulation in Activities of Daily Living for individuals living with mild-to-moderate dementia (CS-ADL): a feasibility study
Source: HRB Open Res. 2026 Oct 15;9:59. Originally published 2026 Jun 2. [Version 2] doi: 10.12688/hrbopenres.14464.2 (PMC13694920; doi:10.12688/hrbopenres.14464.2)
Supplement: Peer review history

# Reviewer report: Reviewer response for version 2

**DOI:** 10.21956/hrbopenres.16066.r58641

**Published:** 2026-10-26

**Authors:** Aniek Puspitosari

**Affiliations:** 1 Poltekkes Kemenkes Surakarta, Jawa Tengah, Indonesia

This manuscript reports a feasibility study of Cognitive Stimulation in Activities of Daily Living (CS-ADL), an occupational therapist-led group intervention designed for people living with mild-to-moderate dementia. The study aimed to assess the feasibility of the intervention and the proposed trial methodology, while also exploring preliminary changes in activities of daily living (ADL) functioning, cognition, communication, quality of life, neuropsychiatric symptoms, and occupational performance. The study enrolled 16 participants, with 13 allocated to CS-ADL and three to treatment as usual (TAU). Due to recruitment difficulties and differences in recruitment rates across sites, randomisation was not feasible, and between-group comparisons were not undertaken.

 The topic is relevant to occupational therapy and dementia care, particularly because CS-ADL integrates cognitive stimulation with meaningful everyday activities. The manuscript also identifies practical challenges related to recruitment, intervention delivery, fidelity, outcome measurement, and caregiver involvement. However, several issues concerning numerical consistency, statistical reporting, and interpretation of findings require clarification before the scientific reliability of the reported results can be established.

**Major comments**

**1. Accuracy and consistency of numerical reporting**

 There are several apparent discrepancies between the baseline and follow-up values and the reported mean differences in the results tables. For example, in Table 3, the QOL-AD mean changes from 66.0 at baseline to 64.2 at follow-up, corresponding to a difference of −1.8, whereas the table reports a mean difference of 0.18 and presents the same confidence interval as the ADCS-ADL outcome. Similarly, the reported baseline and follow-up means for RBANS attention, RBANS visuospatial/constructional ability, and OTTOS do not appear to correspond to their reported mean differences.

 In Table 4, the QOL-AD score changes from 84 to 81, which represents a difference of −3 rather than −4. The HCS score changes from 6 to 10, representing an increase of 4 points rather than the reported −4 points. As higher HCS scores indicate greater communication deficits, the direction of this change also requires clarification.

 The authors should verify all reported values against the original dataset and statistical output, correct any transcription or calculation errors, and ensure that the tables, results narrative, abstract, and conclusions are fully consistent. The reported confidence intervals should also be checked against the corresponding estimates and analysis.

**2. Recruitment, retention, and participant flow**

 The manuscript reports that 16 participants were enrolled and 12 completed follow-up assessments. However, the reported overall dropout rate of 6.25% does not appear consistent with these figures. If four of the 16 enrolled participants did not complete follow-up, the corresponding proportion would be 25%. The authors should clarify the denominator and definition used for the dropout rate and reconcile this figure with the participant flow diagram, abstract, and results section. Recruitment, withdrawal, loss to follow-up, and intervention discontinuation should be distinguished clearly.

**3. Statistical analysis and interpretation**

 The Methods section describes the use of descriptive statistics and assessment of normality, whereas the Results section reports mean differences with 95% confidence intervals and describes a reduction in caregiver distress as significant. The statistical procedures used to derive these estimates and support this interpretation are not sufficiently clear.

 The authors should specify the analysis used for each reported outcome, including how changes and confidence intervals were calculated, the number of participants included in each analysis, and how missing observations were handled. The statement regarding statistical significance should be supported by the reported analysis and interpreted cautiously. Given the small sample and the primary feasibility objectives, the emphasis should remain on feasibility indicators, estimation, and uncertainty rather than claims of intervention effectiveness.

**4. Alignment between the feasibility design and study conclusions**

 The non-randomised design and the substantial imbalance between the intervention and control groups are understandable in the context of the recruitment challenges described. Nevertheless, the study cannot provide a reliable comparative estimate of the effect of CS-ADL relative to TAU. The authors appropriately acknowledge some of these limitations, but the implications should be maintained consistently throughout the manuscript, including the abstract and conclusions.

 The reduction in caregiver distress and the observed changes in cognitive outcomes should be presented as preliminary descriptive findings rather than evidence that the intervention is effective. The authors should also distinguish the feasibility outcomes from the exploratory clinical outcomes and clarify which feasibility criteria were met, not met, or could not be adequately evaluated.

**5. Reproducibility and methodological transparency**

 The intervention description and outcome-measure descriptions provide useful information. However, additional clarification is needed regarding the analysis procedures, the handling of incomplete outcome data, and the availability of the data underlying the reported results. The authors should provide an appropriate data availability statement and indicate whether anonymised data and relevant analysis materials can be accessed, subject to ethical and data-protection requirements.

**Minor comments**

1. The abstract should clearly distinguish the feasibility findings from the exploratory outcome findings and ensure that all numerical statements agree with the corrected results.
2. The manuscript would benefit from a final editorial review to correct typographical errors, grammatical issues, and inconsistencies in terminology and formatting.
3. The Discussion should continue to acknowledge the limitations of the non-randomised design, the small sample, the unequal group sizes, and the lack of blinded outcome assessment when interpreting the findings.

**Overall assessment**

 This study addresses a relevant question for occupational therapy practice and offers useful insights into the practical challenges of evaluating a group-based cognitive stimulation intervention for people living with dementia. The feasibility focus is appropriate, and the implementation findings may inform future research. However, the numerical discrepancies in the outcome tables and participant-flow reporting, together with insufficiently specified statistical procedures, need to be addressed before the results can be interpreted confidently.

 I recommend **major revision**. The essential requirements are to verify and correct the numerical results, reconcile recruitment and retention figures, clarify the statistical methods, and ensure that all interpretations and conclusions remain proportionate to the feasibility design and the limitations of the available data.

Is the work clearly and accurately presented and does it cite the current literature?

Partly

If applicable, is the statistical analysis and its interpretation appropriate?

No

Are all the source data underlying the results available to ensure full reproducibility?

Partly

Is the study design appropriate and is the work technically sound?

Partly

Are the conclusions drawn adequately supported by the results?

Partly

Are sufficient details of methods and analysis provided to allow replication by others?

Partly

Reviewer Expertise:

Occupational Therapy; Gerontology; Activities of Daily Living (ADL); Rehabilitation; Functional

I confirm that I have read this submission and believe that I have an appropriate level of expertise to state that I do not consider it to be of an acceptable scientific standard, for reasons outlined above.

# Reviewer report: Reviewer response for version 2

**DOI:** 10.21956/hrbopenres.16066.r58553

**Published:** 2026-10-23

**Authors:** Ebow Nketsiah

**Affiliations:** 1 a Saint Louis School of University, Saint Louis, Missouri, USA; 2 Sandler School of Social Work, Florida Atlantic University (Ringgold ID: 1782), Boca Raton, Florida, USA

Great job on the revisions!

Is the work clearly and accurately presented and does it cite the current literature?

Yes

If applicable, is the statistical analysis and its interpretation appropriate?

Yes

Are all the source data underlying the results available to ensure full reproducibility?

Yes

Is the study design appropriate and is the work technically sound?

Yes

Are the conclusions drawn adequately supported by the results?

Yes

Are sufficient details of methods and analysis provided to allow replication by others?

Yes

Reviewer Expertise:

Aging and health; dementia and Alzheimer's disease treatments

I confirm that I have read this submission and believe that I have an appropriate level of expertise to confirm that it is of an acceptable scientific standard.

# Reviewer report: Reviewer response for version 2

**DOI:** 10.21956/hrbopenres.16066.r58552

**Published:** 2026-10-16

**Authors:** Michelle Kelly

**Affiliations:** 1 National College of Ireland, Dublin, Leinster, Ireland

Sincere thanks to the authors for the detailed reply and careful attention given to revising the manuscript. This work is so important, and the benefits and challenges described here will be incredibly helpful for others pursuing similar work. We have experienced similar timeline challenges regarding ethics, so I fully empathize. The work completed across the short study timeline is to be commended. 

 I have one very minor suggested edit: for the Abstract Methods, I recommend removing the first sentence "This feasibility study used a non-randomised design" - you go on to explain that randomisation was not possible further down, so that's covered. Currently, it just reads a little odd that the last sentence in the background says "This study investigated the feasibility of... a randomised controlled design" - which is immediately followed by "This feasibility study used a non-randomised design". 

 Otherwise great work. I won't let that one comment hold up approval. 

 Best of luck with further implementation of CS-ADL.

Is the work clearly and accurately presented and does it cite the current literature?

Yes

If applicable, is the statistical analysis and its interpretation appropriate?

Not applicable

Are all the source data underlying the results available to ensure full reproducibility?

Partly

Is the study design appropriate and is the work technically sound?

Partly

Are the conclusions drawn adequately supported by the results?

Yes

Are sufficient details of methods and analysis provided to allow replication by others?

Partly

Reviewer Expertise:

Cognitive stimulation therapy, cognitive rehabilitation,  implementation research, behavioural psychology

I confirm that I have read this submission and believe that I have an appropriate level of expertise to confirm that it is of an acceptable scientific standard.

# Reviewer report: Reviewer response for version 1

**DOI:** 10.21956/hrbopenres.15938.r57067

**Published:** 2026-09-16

**Authors:** Michelle Kelly

**Affiliations:** 1 National College of Ireland, Dublin, Leinster, Ireland

Thank you for the opportunity to review this interesting paper on cognitive stimulation in activities of daily living for individuals living with mild to moderate dementia. This is important work, particularly as there tends to be low transfer of effects from general cognitive training to activities of daily living. I commend the authors for their work on this. While the paper is interesting and well written, major revision is required before it can be considered for indexation.

**Overall Study Design/Approach:**

 There has been a significant amount of work in recent years focusing on clearly defining the design and parameters of feasibility (and pilot) studies. This has been of great benefit to supporting improved quality and reporting of these types of studies. Here, the research is described as a feasibility study, yet pre and post intervention outcomes are analysed, power analysis included etc. I would like the authors to consider, clearly define, and justify the design of the research, taking into account their previous pilot study. Consider all aspects of the written manuscript, what constitutes a feasibility study, and what may require revision. This would significantly strengthen the paper. For this, please carefully read the following papers:

 [Ref 1]

 [Ref 2]

 [Ref 3]

 Also, a possibly helpful sample of a feasibility study is here; [Ref 4]

 All relevant sections of the manuscript should be revised based on the considerations above.

**Introduction:**

 Well written with a valuable discussion present, particularly in drawing attention to the relevance of cognitive stimulation interventions for occupational therapy and to the importance of functional outcomes for people living with dementia. However, I suggest that the framing is somewhat refined so that CST is not implicitly evaluated against an outcome it was not primarily designed to address. As the authors note, the most consistently targeted outcomes of CST are global cognition and quality of life, with some evidence relating to language, communication and depressive symptoms. Although ADL and IADL outcomes are assessed in some CST studies, these are generally secondary (or “downstream”) outcomes rather than the principal therapeutic targets of CST. Consequently, inconsistent evidence concerning ADL outcomes does not necessarily indicate that CST is failing to achieve its intended purpose. The occupational therapy perspective remains important, and the manuscript could instead frame the issue as a question of whether, and under what circumstances, CS(T) might also support occupational engagement or functional performance. This would preserve the value of examining CST’s applicability to occupational therapy without implying that CST was originally intended to produce direct improvements in ADLs.

 The relationship between two of the cited reviews could also be reconsidered. Woods et al. (2023) considered cognitive stimulation broadly, including CST alongside reality orientation, individual interventions and multicomponent approaches. Ryan and Brady (2023) also appear to examine varied formats of cognitive stimulation targeting functional outcomes. Is there overlap in some of the scope, aims or included interventions of these two reviews? If so, the findings might be framed as complementary or as Ryan and Brady extending and refining the broader evidence identified by Woods et al., rather than suggesting that one addresses limitations of the other. This clarification would strengthen the rationale for the research and help readers to understand the distinct contribution.

 Minor point: CST has 18 key principles (as opposed to 8).

**Methods: **Revise as described in point 1.

 Other points: Julius & Owen (2006) do not provide recommendations for sample size of pilot and feasibility studies – they provide a methodology for calculating sample sizes in full scale clinical trials when the sample variance has less than 200 degrees of freedom. Take care with citations to ensure accuracy.

 Consent - Were the participants given an opportunity to contact the researchers to ask questions about the research; Were there other formats offered in terms of providing information about the study (E.g. a phone call); Was there PPI guidance on writing information sheets and consent forms; Were those familiar with prospective participants consulted to help to ascertain capacity; Was continued assent assessed? Of course, we must assume people have capacity until the contrary is shown, but we also must demonstrate that supports have been included to ensure genuine, informed consent has been obtained. I’m sure the ethics committee addressed all of this but the description in text makes it seem as though there was no additional support offered to ensure that where consent was provided, that it was fully informed. The text also may imply that the capacity act supports the idea that researchers simply send written information/consent forms, and if they are signed, we proceed without question. This just undermines the likely care and attention that was given to your participants, so add further clarification here.  

 The CS-ADL format is one session or theme per week for seven weeks, which is a little different to the format of CST, which is two sessions per week for 7 weeks (although some are now incorporating two sessions in one day). If you wish to use CST as the basis for comparison, you can clarify this when comparing formats (e.g., in the introduction). Also, CST stipulates the requirement for two facilitators at all sessions.

 TAU needs to be described in more detail. What did this treatment entail? This could also help us to understand why only three people were ultimately suitable for inclusion in the TAU group.

 Additional information would be helpful to explain why randomisation was not feasible. Of course, recruitment difficulties are common in feasibility studies, but slow or unequal recruitment across sites does not make individual randomisation impossible. Participants can generally be randomised as they are recruited, using stratification by site or a central allocation procedure. Small numbers may produce imbalance, but the objective here is to test whether randomisation/study procedures can work (obtaining adequately powered comparisons is not needed for a feasibility study).

 I appreciate the content on intervention integrity, and would like more information as to how this was assessed, did the researchers use IOA or IRR data, checklists, direct observation, etc?  Also on that section, was the reality orientation included to maintain consistency with CST? Given the participants feedback, it would be of interest to mention whether the CST key principle of “Using orientation sensitively and implicitly” was incorporated.

**Discussion:**

 The discussion is well written and considered. It would be great to see you include your prior pilot as part of the discussion here also. If you proceed with the feasibility approach, the discussion of power, study outcomes/pre-post analysis, etc can be reconsidered.

Is the work clearly and accurately presented and does it cite the current literature?

Yes

If applicable, is the statistical analysis and its interpretation appropriate?

Not applicable

Are all the source data underlying the results available to ensure full reproducibility?

Partly

Is the study design appropriate and is the work technically sound?

Partly

Are the conclusions drawn adequately supported by the results?

Yes

Are sufficient details of methods and analysis provided to allow replication by others?

Partly

Reviewer Expertise:

Cognitive stimulation therapy, cognitive rehabilitation,  implementation research, behavioural psychology

I confirm that I have read this submission and believe that I have an appropriate level of expertise to confirm that it is of an acceptable scientific standard, however I have significant reservations, as outlined above.

## References

[1] : Role of feasibility and pilot studies in randomised controlled trials: a cross-sectional study. *BMJ Open* .2018;8(9) : 10.1136/bmjopen-2018-022233 10.1136/bmjopen-2018-022233 PMC616976230257847

[2] : Defining Feasibility and Pilot Studies in Preparation for Randomised Controlled Trials: Development of a Conceptual Framework. *PLOS ONE* .2016;11(3) : 10.1371/journal.pone.0150205 10.1371/journal.pone.0150205 PMC479241826978655

[3] : Pilot and feasibility studies: extending the conceptual framework. *Pilot and Feasibility Studies* .2023;9(1) : 10.1186/s40814-023-01233-1 10.1186/s40814-023-01233-1 PMC990998536759879

[4] : Group cognitive stimulation therapy for people with intellectual disability and dementia: feasibility randomised controlled trial. *BJPsych Open* .2025;11(5) : 10.1192/bjo.2025.10764 10.1192/bjo.2025.10764 PMC1234442640744455

# Response

**Published:** 2026-10-05

**Authors:** Simone Ryan

**Affiliations:** School of Allied Health, University of Limerick Faculty of Education and Health Sciences, Limerick, County Limerick, Ireland

Dear Dr. Kelly, 

 Thank you for your detailed and insightful review of our paper, we appreciate the time spent on providing these comments and feel that subsequent changes have strengthened the quality of our reporting. Below are my responses to your comments: 

 1. **Overall study design/approach**: Thank you for highlighting this concern regarding the reporting of our study design. We have added further detail to the revised manuscript regarding the rationale for the feasibility design.

 We have highlighted that this study design was chosen as no prior studies had evaluated the current CS-ADL intervention with regards to optimal content and delivery, with feasibility testing required as it was anticipated that further intervention refinements would be required prior to future pilot trials and larger scale evaluation.

 In reference to the previous pilot study completed, it has been clarified that any prior 'piloting' of CS-ADL involved service-level testing of a sample format of the intervention, evaluated qualitatively. Further intervention refinements had been made since this testing and the current study thus evaluates the updated intervention.

 While this study reported pre- and post-intervention outcomes, we have clarified the rationale for this in the revised manuscript. Hypothesis testing was not an objective of this study, and therefore formal power calculations have been retracted, yet it is highlighted that evaluation of intervention outcomes is an important part of feasibility study to evaluate chosen measurement tools and the potential for the intervention to affect outcomes in future trials. 

 2. **Introduction: **Thank you for highlighting the concern relating to the framing of CST's relevance within occuaptional therapy and functional outcomes. The manuscript has since been revised to reframe the discussion of CST. It has been highlighted that while the primary outcome of CS is to enhance cognitive and social functioning, CST is delivered by OTs as a means through which to remediate underlying impaired cognitive functions that impede ADL performance, through a 'bottom-up' intervention approach. Existing evidence has highlighted the effectiveness for improving its intended primary outcomes, with applications of CST to other secondary outcomes requiring further investigation. 

 The relationship between the Woods et al. (2023) and the Ryan & Brady (2023) reviews has also been revised to highlight their differing objectives and complementary findings. 

 The manuscript has also been revised to update the number of guiding principles for CST from eight to eighteen. 

 3. **Methods:** Thank you for highlighting this oversight in relation to the Julius & Owen (2006) citation. This citation has since been removed, with sample size rationale updated in line with more recent literature. 

 With regards to consenting procedures, further detail has been provided in the revised manuscript regarding the support provided to prospective participants in the decision-making process. Yes, gatekeppers familiar with the prospective participants provided study information verbally, and enquired whether participants wished to contact the researcher themselves, or whether they would prefer for the researcher to contact them directly. For the latter, verbal consent was obtained for participant contact details to be provided to the researcher for the sole purpose of providing further study information. Study information was initially provided verbally over phone call, and participant information leaflets were thus provided to support the decision-making process, with the individual’s caregiver informed of the research where possible. All participant-facing documentation followed dementia-friendly document guidelines. Continued assent was not formally assessed; however, the researcher informally assessed verbal and non-verbal participant cues throughout the study and ensured to continually inform participants and caregivers that they could withdraw from the study at any stage prior to data anonymisation. 

 The introduction has also been updated to highlight that practical real-world implementation of CST often differs from the prescribed format of two sessions per week, with the two 45-minute sessions often incorporated into one 2-hour session delivered once weekly due to service and resource constraints. 

 TAU has been described in more detail in the revised manuscript. Participants received TAU at their respective clinical setting where recruitment took place and consisted of the routine care that participants received from the health services. TAU may have differed between participants and was determined by healthcare providers who are external to the research. It may have included medication, occupational therapy, physical therapy, nursing or attendance at a day care centre. It may also have consisted of no active treatment or service. It was not possible for participants in the control to receive CS-ADL as part of their usual care as it was not implemented in the services where participants were recruited from.

 Additional information regarding challenges implementing intended randomisation procedures has been highlighted in the discussion section. It was initially intended to randomise participants to treatment and control conditions within each distinct site. However, insufficient participants were recruited at each site for randomisation to be undertaken, as CS-ADL is a group intervention and required a minimum number of participants for the intervention to be delivered. Cluster randomisation was considered as an alternative randomisation procedure, where study sites would be randomised to treatment conditions, as opposed to individual participants. However, this was ultimately not feasible in the current study, due to differing rates of recruitment at each planned cluster site and time constraints. Instead, participants were non-randomly allocated depending on their intervention site. Due to aforementioned differing rates of recruitment, this resulted in low numbers within the control group.

 Regarding intervention fidelity, no standardised checklists or direct observation were used, which is a noted limitation of the study. Instead, an intervention manual was developed by the research team to support standardised delivery across sites; any deviations from the intervention manual were recorded to capture intervention integrity and were reflected on after each intervention session.

# Reviewer report: Reviewer response for version 1

**DOI:** 10.21956/hrbopenres.15938.r56843

**Published:** 2026-08-12

**Authors:** Ebow Nketsiah

**Affiliations:** 1 a Saint Louis School of University, Saint Louis, Missouri, USA; 2 Sandler School of Social Work, Florida Atlantic University (Ringgold ID: 1782), Boca Raton, Florida, USA

This is an important and timely study, particularly given the growing need for effective interventions for people living with dementia. The authors have done an excellent job establishing the rationale for the study and clearly articulating its significance within the broader context of dementia treatment research.

 As acknowledged by the authors, the challenges encountered throughout the study inevitably limit the interpretation of the findings and the strength of the conclusions that can be drawn. While it would have been encouraging to observe statistically significant outcomes across the intervention measures, I commend the authors for reporting these findings transparently. The study provides valuable preliminary evidence and offers important insights that can inform the design and implementation of future intervention research.

 I have a few suggestions that may further strengthen the manuscript:

1. **Reliability of outcome measures:** Consider reporting the reliability (e.g., Cronbach's alpha or other relevant reliability estimates) of the outcome measures used in this study within the Methods section. This would provide readers with additional information regarding the psychometric properties of the instruments.
2. **Description of outcome measures:** It would also be helpful to include a brief description of each outcome measure, including its purpose, scoring procedure, possible score range, and interpretation of scores. This information will improve the readability of the Methods section and help readers better understand the reported findings.
3. **Clarification of the CS-ADL intervention:** In the intervention integrity section, the manuscript cites Spector et al. (2020) in relation to CS-ADL. However, it is not entirely clear how CS-ADL differs from, or relates to, standard Cognitive Stimulation Therapy (CST). Providing a brief explanation of the relationship between the two interventions would improve clarity. If CS-ADL represents an adaptation or modification of CST, it would be helpful to describe the specific modifications that were made and the rationale for those changes.

Overall, I believe this manuscript makes a meaningful contribution to the growing literature on non-pharmacological interventions for dementia. With these minor revisions, it will be even stronger and more informative for readers and researchers in the field.

Is the work clearly and accurately presented and does it cite the current literature?

Yes

If applicable, is the statistical analysis and its interpretation appropriate?

Yes

Are all the source data underlying the results available to ensure full reproducibility?

Yes

Is the study design appropriate and is the work technically sound?

Yes

Are the conclusions drawn adequately supported by the results?

Yes

Are sufficient details of methods and analysis provided to allow replication by others?

Yes

Reviewer Expertise:

Aging and health; dementia and Alzheimer's disease treatments

I confirm that I have read this submission and believe that I have an appropriate level of expertise to confirm that it is of an acceptable scientific standard, however I have significant reservations, as outlined above.

# Response

**Published:** 2026-10-05

**Authors:** Simone Ryan

**Affiliations:** School of Allied Health, University of Limerick Faculty of Education and Health Sciences, Limerick, County Limerick, Ireland

Dear Dr. Nketsiah, 

 Thank you for the time taken to provide this review for our paper, we appreciate the important comments that we believe have strenghtened the quality of our reporting and clarity for the readers. Please see below my responses to the comments:

 1. **Reliability of outcome measures:** Thank you for highlighting this important omission from the original manuscript. The manuscript has since been revised to include measures of reliability and other psychometric properties where the information was available. 

 2. **Description of outcome measures:** The maunscript has been revised to include a brief description of each outcome emasure, including its purpose, scoring procedure, score range and interpretation to improve the readability of the methods section. 

 3. **Clarification of the CS-ADL intervention:** CS-ADL primarily differs from CST with regards to its focus on ADL practice and ADL-themed cognitive stimulation activities. This has been clarified in the revised manuscript.
